# New Insights into the Phytochemical Profile and Biological Properties of *Lycium intricatum* Bois. (Solanaceae)

**DOI:** 10.3390/plants12050996

**Published:** 2023-02-22

**Authors:** Houaria Bendjedou, Houari Benamar, Malika Bennaceur, Maria João Rodrigues, Catarina Guerreiro Pereira, Riccardo Trentin, Luísa Custódio

**Affiliations:** 1 Faculty of Natural Sciences and Life, Department of Biology, University of Oran1, El M’Naouer, P.O. Box 1524, Oran 31000, Algeria; 2 Laboratory of Research in Arid Areas, University of Science and Technology Houari Boumediene, P.O. Box 32, Algiers 16111, Algeria; 3 Centre of Marine Sciences (CCMAR), Faculdade de Ciências e Tecnologia, Universidade do Algarve, Ed. 7, Campus de Gambelas, 8005-139 Faro, Portugal; 4 Department of Biology, University of Padova, Via U. Bassi, 58/B 35131 Padova, Italy

**Keywords:** medicinal plants, phenolic compounds, oxidative stress, neuroprotection, diabetes, tyrosinase, goji

## Abstract

This work aimed to boost the valorisation of *Lycium intricatum* Boiss. *L.* as a source of high added value bioproducts. For that purpose, leaves and root ethanol extracts and fractions (chloroform, ethyl acetate, *n*-butanol, and water) were prepared and evaluated for radical scavenging activity (RSA) on 2,2-diphenyl-1-picrylhydrazyl (DPPH) and 2,2′-azino-bis (3-ethylbenzothiazoline-6-sulfonic acid) (ABTS) radicals, ferric reducing antioxidant power (FRAP), and metal chelating potential against copper and iron ions. Extracts were also appraised for in vitro inhibition of enzymes implicated on the onset of neurological diseases (acetylcholinesterase: AChE and butyrylcholinesterase: BuChE), type-2 diabetes *mellitus* (T2DM, α-glucosidase), obesity/acne (lipase), and skin hyperpigmentation/food oxidation (tyrosinase). The total content of phenolics (TPC), flavonoids (TFC), and hydrolysable tannins (THTC) was evaluated by colorimetric methods, while the phenolic profile was determined by high-performance liquid chromatography, coupled to a diode-array ultraviolet detector (HPLC-UV-DAD). Extracts had significant RSA and FRAP, and moderate copper chelation, but no iron chelating capacity. Samples had a higher activity towards α-glucosidase and tyrosinase, especially those from roots, a low capacity to inhibit AChE, and no activity towards BuChE and lipase. The ethyl acetate fraction of roots had the highest TPC and THTC, whereas the ethyl acetate fraction of leaves had the highest flavonoid levels. Gallic, gentisic, ferulic, and trans-cinnamic acids were identified in both organs. The results suggest that *L. intricatum* is a promising source of bioactive compounds with food, pharmaceutical, and biomedical applications.

## 1. Introduction

Medicinal herbs contain different phytochemicals, with a broad spectrum of pharmacological effects, that have already proved to be effective therapeutic tools in the treatment of several diseases. For example, different flavonoids and other phenolic compounds display strong antioxidant activities and inhibitory properties against enzymes involved in human ailments, such as acetylcholinesterase (AChE) and butyrylcholinesterase (BuChE), which are involved in the onset of Alzheimer’s disease (AD) and other neurodegenerative disorders, and α-glucosidase, linked with type-2 diabetes *mellitus* (T2DM) [1,2].

The genus *Lycium* (Solanaceae) comprises about 80 species distributed worldwide [3]. Algeria has four species, namely *L. arabicum* Boiss., *L. europaeum* L., *L. halmifolium* Mill., and *L. intricatum* Boiss., which are mainly distributed in the north [4]. Species belonging to the genus *Lycium*, especially *L. barbarum* L. and *L. chinense* Mill., have been an important source of traditional remedies against a high number of human diseases, including AD, diabetes, obesity, and cancer, and of nutritional supplements in Southeast Asia, mostly in China [5,6,7,8]. The interest in *Lycium* fruits, known as goji, has increased tremendously in Western countries, due to its nutritional properties (e.g., proteins, amino acids, and vitamins) and the presence of bioactive compounds (e.g., phenolics, flavonoids, and anthocyanins), with functional properties (e.g., antioxidant, anti-inflammatory) which confers goji a plethora of health promoting functions, such as, for example, anti-aging and anti-diabetic [9]. In fact, goji berries are considered a functional food, and the global distribution and diverse uses make *Lycium* a genus of global importance. Goji and other *Lycium* parts, such as leaves, seeds, and flowers, display substantial biological activities, like immunomodulation, retinal protection, anti-tumour, hypotensive, neuroprotective, anti-diabetic, skin care, enzyme inhibition, and antioxidant, linked with their chemical composition that include polyphenols, alkaloids, and sesquiterpenes [3,6,10,11]. For example, goji leaves have a chemical composition like berries, with reduced levels of sugars and a higher abundance of fibres [12], and are rich in bioactive metabolites (e.g., phenolic compounds and alkaloids) and present important biological activities, including antioxidant, anti-inflammatory, and anti-diabetic [12].

Research has mainly focused on *L. barbarum* and *L. chinense* [12], but other *Lycium* species may hold potential as sources of high added value products. *Lycium intricatum* Boiss., also called “Awsadj”, is a spiky shrub that can reach 3 m high, with fleshy fruits with a red colour, when mature. In Algeria, it inhabits maritime rocks and arid lands on the littoral [4,6]. In traditional medicine, a decoction of the leaves is made twice, left to cool for one day, and then applied in drops for cataracts and eye inflammations [13]. The seeds are used for helminthiasis and as a digestive, while fruits are used for the treatment of eye diseases [14]. Several bioactive molecules were previously identified in different organs of *L. intricatum*. For example, fatty acids, such as myristic, palmitic, palmitoleic, oleic, linoleic, and erucic acids, and sterols like ergosterol, stigmasterol, and β-sitosterol, and triterpenes like squalene, erythrodiol, and uvaol, were identified in the seeds [15]. One phenolic acid, eight phenolic acid derivatives, and six flavonoids were identified in leaves and fruits [16], and one new ionone derivative and three known compounds, namely isoscopoletin, 3,4,5-trimethoxybenzyl alcohol, and (+)-isolariciresinol, were isolated and identified in leaves [17]. To our best knowledge, only one paper has described biological activities of *L. intricatum*, focusing on the antioxidant activity of the methanol extract of leaves and fruits by complementary methods, namely radical scavenging properties towards 2,2-diphenyl-1-picrylhydrazyl (DPPH), 2,2′-azinobis (3-ethylbenzothiazoline-6-sulphonic acid diammonium salt) (ABTS), hydroxyl free radicals, and ferric-reducing antioxidant power (FRAP) [16]. In that work, leaves exhibited the upmost antioxidant potential, coupled to the highest levels of phenolics and flavonoids, leading the authors to conclude that *L. intricatum* should be further explored as a potential source of high added value bioactive products [16]. Presumably, there are no reports of the biological properties of the roots of this species.

*Lycium intricatum* is, therefore, considered an underexploited species, despite its high potential to serve as a source with economic and nutritional value [15]. Providing better information regarding the chemical composition and pharmacological properties of this species would pave the way to its valorisation as a source of bioactive compounds, and consequently, to agriculture and economic progress [15]. In this context, in the present work, qualitative and quantitative analyses of the phenolic composition of an ethanol crude extract and obtained fractions of roots and leaves of this species were performed by colorimetric methods and high-performance liquid chromatography, coupled to a diode-array ultraviolet detector (HPLC-UV-DAD). The extracts were also evaluated for in vitro antioxidant capacity, by complementary assays, and for enzymatic inhibitory properties toward enzymes related with the onset of AD (AChE and BuChE), T2DM (α-glucosidase), obesity/acne (lipase), and skin hyperpigmentation/food oxidation (tyrosinase).

## 2. Materials and Methods

### 2.1. Chemicals and Reagents

All the chemicals used in this work were of analytical grade. Sigma-Aldrich (Lisbon, Portugal) supplied Folin-Ciocalteau (F-C) phenol reagent, sodium acetate, sodium nitrite, DPPH, ABTS, ascorbic acid, butylated hydroxytoluene (BHT), AChE (from electric eel, Type-VIS, EC 3.1.1.7), BuChE (from horse serum, EC 3.1.1.8), acetylthiocholine iodide, butyrylthiocholine chloride, galantamine hydrobromide (from *Lycoris* sp.), α-glucosidase (from yeast, *Saccharomyces cerevisiae*, EC 3.2.1.20), 4-nitrophenyl-α-D-glucopyranoside, acarbose, lipase from porcine pancreas (Type II, EC 3.1.1.3), orlistat, tyrosinase (from mushroom, EC 1.14.18.1), L-2,3-dihydroxyphenylalanine, arbutin, and phosphate buffer. Ethylenediaminetetraacetic acid (EDTA) was obtained from VWR (Carnaxide, Portugal). Additional reagents and solvents were obtained from Merck (Lisbon, Portugal).

### 2.2. Plant Material

Roots and leaves from L. intricatum plants were harvested in 2018, in Ain El Turk, Oran, Algeria (35°44′16.7″ N, 0°43′30.5″ W, 66 m a.s.l.) during the flowering season (May). The plant was identified by Prof. Abderrazak Marouf, Institute of Science and Technology, University Centre of Naama, Naama, Algeria. A voucher specimen (OUE.2018.C1) was deposited in the Department of Biology, University of Oran1, Oran, Algeria. The roots and leaves were dried in a well-ventilated room at 30 °C for 72 h, fully grinded, and stored in the dark at room temperature (RT) until use.

### 2.3. Extraction and Partition

Dried samples (200 g) were extracted by cold maceration, three times with ethanol, (1.2 L) for 72 h at RT. The extracts were filtered through Whatman N°1 filter paper, combined, and the solvent was removed under reduced pressure at 40 °C. The crude extract (12 g) was dissolved in distilled water (240 mL) and sequentially extracted with chloroform (240 mL *×* 3), ethyl acetate (240 mL *×* 3), and *n*-butanol saturated with water (240 mL *×* 3). Obtained fractions were dried in a rotary evaporator, as previously described, for the crude extract. The crude extract and obtained fractions were resuspended in methanol, at a concentration of 10 mg/mL, and stored at −20 °C until use.

### 2.4. Total Contents of Phenolics (TPC), Flavonoids (TFC), and Hydrolysable Tannins (THTC)

TPC was evaluated by the F-C assay with absorbance measured at 760 nm. Gallic acid was used as standard, and results were expressed as milligrams of gallic acid equivalents per gram of dried extract (mg GAE/g DE). TFC was determined by the aluminium chloride colorimetric assay, the absorbance was measured at 510 nm using catechin as standard, and results were expressed as milligrams of catechin equivalents per gram of dried extract (mg CE/g DE). All methods are detailed in [18,19]. THTC were determined using potassium iodate assay, the absorbance was measured at 550 nm using tannic acid, as standard, and results were expressed as milligrams of tannic acid equivalents per gram of dried extract (mg TAE/g DE) [20].

### 2.5. HPLC-UV-DAD Analysis and Identification of Phenolic Compounds

The extracts at the concentration of 10 mg/mL were analysed by HPLC-UV-DAD (Agilent 1200 Series LC system, Waldbronn, Germany), as described elsewhere [21]. For identification of phenolic compounds, the retention parameters of each assay were compared with the standard controls and the peak purity with the UV-vis spectral reference data. Commercial standards of gallic, gentisic, trans-cinnamic, ferulic, and p-coumaric acids, gallocatechin gallate, catechin, rutin, and quercetin were prepared in methanol and analysed separately.

### 2.6. Antioxidant Activity

#### 2.6.1. Radical Scavenging Activity (RSA) on DPPH Radical

Samples were tested for RSA against the DPPH radical at concentrations ranging from 10 to 1000 µg/mL, as described previously [22]. Ascorbic acid was used as a positive control at concentrations ranging from 10 to 500 µg/mL. Results were expressed as percentage of inhibition, relative to a control containing DMSO in place of the sample, and as half effective concentration (EC_50_ values, µg/mL).

#### 2.6.2. RSA on ABTS Radical Cation

The RSA against ABTS**^•+^** was evaluated according to Re et al. [23]. A stock solution of ABTS**^•+^** (7.4 mM) was prepared in potassium persulfate (2.6 mM) and left in the dark for 12–16 h at RT. The ABTS**^•+^** solution was then diluted with ethanol to get an absorbance of 0.7 at 734 nm (Biotek Synergy 4, Biotek, Winooski, VT, USA). Samples (10 µL), at concentrations ranging from 1 to 1000 µg/mL, were mixed with 190 µL of ABTS**^•+^** solution in 96-well microplates, and after 6 min of incubation, the absorbance was measured at 734 nm. Results were presented as antioxidant activity (%), relative to a control containing DMSO, and as EC_50_ values (µg/mL). Ascorbic acid was used as a positive control at concentrations ranging from 10 to 500 µg/mL.

#### 2.6.3. Ferric Reducing Antioxidant Power (FRAP)

The ability of the extracts to reduce Fe^3+^ was assayed by the method described by Rodrigues et al. [22]. Absorbance was measured at 700 nm, and increased absorbance of the reaction mixture indicated increased reducing power. Results were expressed as a percentage, relative to the positive control (BHT, 1 mg/mL), and as EC_50_ values (µg/mL).

#### 2.6.4. Metal Chelating Activity on Iron (ICA) and Copper (CCA)

ICA and CCA were tested on samples at different concentrations (10–4000 µg/mL), as described previously [22]. The change in colour was measured on a microplate reader. EDTA was used as the positive control at concentrations ranging from 10 to 500 µg/mL. Results were expressed as percentage of inhibition, relative to a control containing DMSO in place of the sample, and as EC_50_ values (µg/mL).

### 2.7. Enzyme Inhibitory Assays

#### 2.7.1. AChE and BChE Inhibition Assay

The extracts, at concentrations ranging from 10 to 4000 µg/mL, were evaluated for their inhibitory activity against AChE and BuChE, according to Orhan et al. [24]. Absorbances were read at a wavelength of 412 nm using a 96-well microplate reader, and results were expressed as percent inhibition, relative to a control containing DMSO instead of extract, and as half maximal inhibitory concentration (IC_50_ values) (µg/mL). Galantamine (1 to 1000 µg/mL) was used as a reference.

#### 2.7.2. α-Glucosidase Inhibition Assay

The α-glucosidase inhibitory activity was determined according to the method described by Kwon et al. [25]. The absorbances were recorded at 405 nm in a microplate reader and results were expressed as inhibition (%), related to a control containing DMSO, and as IC_50_ values (µg/mL). Acarbose was used as a positive control at concentrations varying from 10 to 4000 µg/mL.

#### 2.7.3. Lipase Inhibition Assay

The inhibitory activity on lipase was evaluated according to the method described by McDougall et al. [26], adapted to 96-well microplates. Samples (20 μL), at concentrations ranging from 10 to 4000 µg/mL, were mixed with 200 μL of Tris-HCl buffer (100 mM, pH 8.2), 20 μL of the enzyme solution (1 mg/mL), and 20 μL of the substrate (4-nitrophenyl dodecanoate, 5.1 mM in ethanol). After an incubation period of 10 min at 37 °C, the absorbance was read at 410 nm. Orlistat was used as the positive control at concentrations ranging from 10 to 1000 µg/mL. Results, calculated as a percentage of inhibitory activity in relation to a control containing the corresponding solvent, in place of the sample, were expressed as IC_50_ values (µg/mL).

#### 2.7.4. Tyrosinase Inhibition Assay

The extracts’ ability to inhibit tyrosinase was assessed following Custódio et al. [27], using arbutin as a positive control at concentrations ranging from 10 to 1000 µg/mL. The extracts were tested at the concentrations ranging from 10 to 4000 µg/mL. The results were calculated and expressed, as in Section 2.7.3.

### 2.8. Statistical Analysis

All the tests were carried out in triplicate. Results were expressed as mean ± standard error mean (SEM). Statistical analysis was performed by one-way analysis of variance (ANOVA), followed by Tukey and Student–Newman–Keuls post hoc test for multiple comparisons. Statistical analysis was performed by using IBM SPSS statistics V24 software from IBM. A value of *p* < 0.05 was considered to indicate statistical significance.

## 3. Results and Discussion

### 3.1. Phenolic Composition of the Extracts

Results on the extraction yields and total contents of phenolics, flavonoids, and tannins are summarized in Table 1. The extraction yield of the crude ethanol extracts was higher for leaves (11.07%) than for roots (1.805%). As a result, the extraction yields of the fractions made from the ethanol extract from leaves (range: 0.118–3.873%) were higher than their counterparts obtained from roots (range: 0.021–0.463). Phenolics have recognized benefits on human health, including antioxidants and enzyme inhibitors [28]. Having this in mind, the extracts were evaluated for their total content in different phenolic groups, and results are depicted in Table 1.

Root extracts had a higher content of phenolics than leaves, with TPC in the following order: ethyl acetate fraction ≥ *n*-butanol fraction > ethanol extract ≥ chloroform fraction > water fraction. In roots, flavonoids peaked in the ethyl acetate fraction, followed by the *n*-butanol one. Finally, high levels of tannins were detected in the root’s ethyl acetate and *n*-butanol fractions, as well as in the ethyl acetate fraction from leaves. In fact, we observed that the ethyl acetate and the *n*-butanol fractions have a higher concentration of total phenolics, flavonoids, and tannins when compared to the ethanol crude extract, probably due to the enrichment in such compounds, due to the higher extractable capacity of such solvents. Similar results were obtained in a related species, *L. europaeum*, by Bendjedou et al. [11]. The obtained results clearly show the influence of the solvent on the extractability of phenolics, flavonoids, and tannins. Phenolic compounds were effectively extracted from the crude ethanol extract, with ethyl acetate and *n*-butanol, whereas chloroform and water allowed for lower amounts of those compounds. In a previous study on the chemical composition of roots and leaves of *L. europaeum* from Algeria, high levels of phenolics, flavonoids, and tannins were also detected in similar extracts [11]. However, lower contents of phenolics and flavonoids were detected in methanol extracts made from leaves and fruits of *L. intricatum* collected from Tunisia [16]. These differences may be related to the solvent used for the extraction and to environmental factors. In effect, the extraction of phenolics is influenced by several conditions, such as the method of extraction, climate, and geographical region of collection, which directly affect the amounts of these molecules in the plant tissues [29]. Phenolic compounds, like those found in high amounts in *L. intricatum*, display important bioactive properties highly relevant for human health improvement, such as anti-inflammatory, anti-anthelmintic, and anti-cataract [30,31,32], which can support the traditional medicinal uses of the plant.

**Figure 1:**
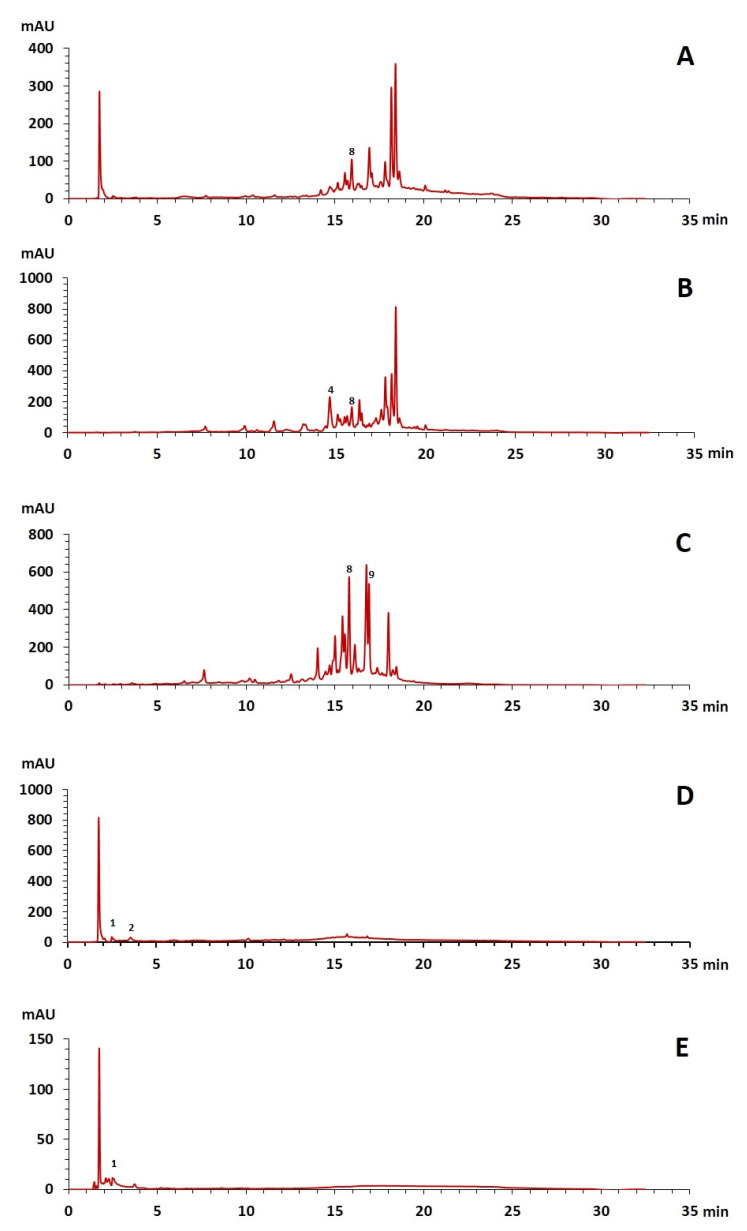
HPLC-DAD-UV analysis (280 nm) of phenolic compounds in the crude ethanol extract (**A**), chloroform (**B**), ethyl acetate (**C**), *n*-butanol (**D**), and water (**E**) fractions of roots of *L. intricatum.* Gallic acid (**1**), gentisic acid (**2**), *p*-coumaric acid (**4**), ferulic acid (**8**), *trans*-cinnamic acid (**9**). The experimental conditions are described in Section 2.5.

**Figure 2:**
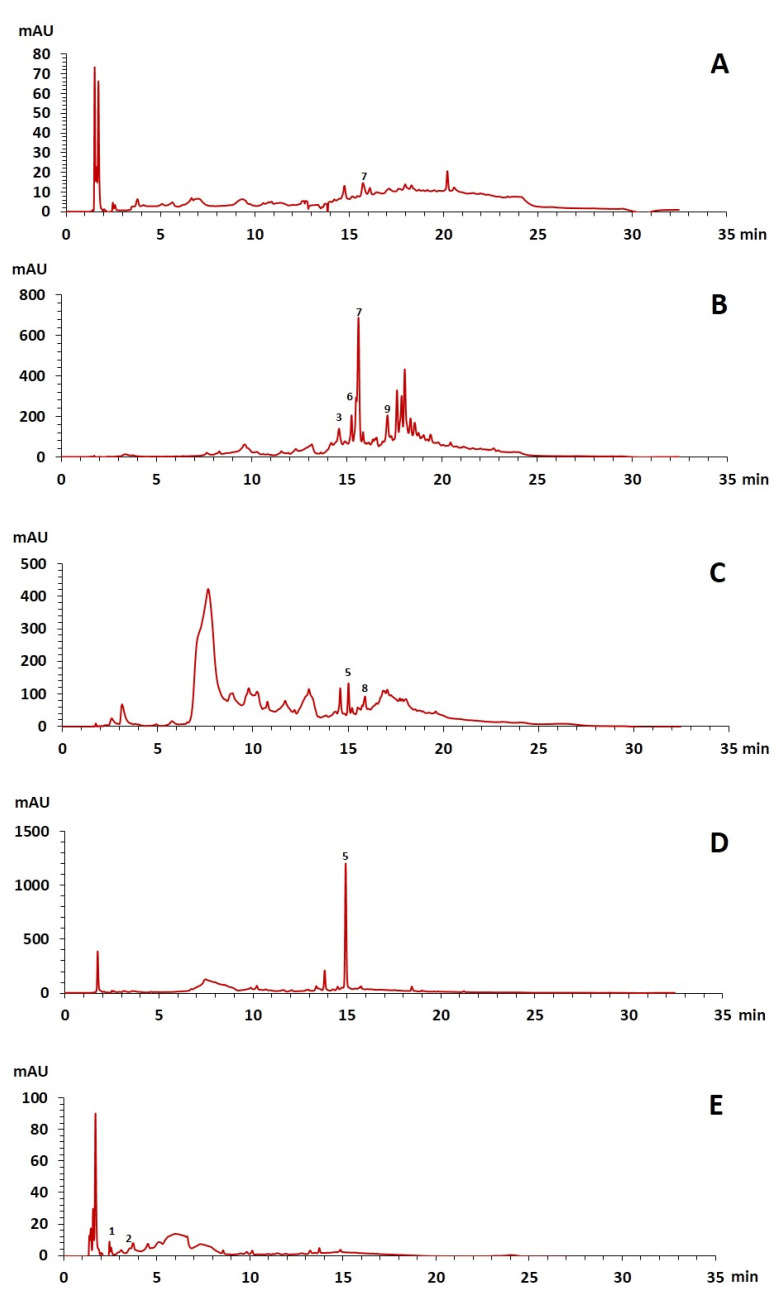
HPLC-DAD-UV analysis (280 nm) of phenolic compounds in ethanol extract (**A**), chloroform (**B**), ethyl acetate (**C**), *n*-butanol (**D**), and water (**E**) fractions of leaves of *L. intricatum.* Gallic acid (**1**), gentisic acid (**2**), catechin (**3**), rutin (**5**), gallocatechin gallate (**6**), quercetin (**7**), ferulic acid (**8**), *trans*-cinnamic acid (**9**). The experimental conditions are described in Section 2.5.

The phenolic composition of the extracts of *L. intricatum* was further investigated through the identification of some individual phenolic compounds by HPLC-UV-DAD, and results are depicted in Figure 1 and Figure 2. Information related to the identified compounds can be found in Table 2. From the twenty-four standards tested, nine compounds were identified in those samples. Among these, five and eight compounds were detected in extracts from roots and leaves, respectively. *p*-coumaric acid (**4**) was specific to roots, while catechin (**3**), rutin (**5**), gallocatechin gallate (**6**), and quercetin (**7**) were preferentially detected in leaves. Gallic (**1**), gentisic (**2**), ferulic (**8**), and *trans*-cinnamic (**9**) acids were identified in both organs. To the best of our knowledge, the presence of compounds **1**–**4** and **6**–**9** in *L. intricatum* is described here for the first time. The detected phenolic compounds are promising nutraceutical and food additives due to their bioactivities, which include inhibition of enzymes involved in generating inflammatory and immune responses (e.g., serine protein kinases, phospholipases, lipoxygenase, cyclooxygenase, and nitric oxide synthase), modulation of glucose and lipid metabolism, and antioxidant, anticancer, and antimicrobial properties [33].

Previous reports indicated the presence of several phenolic compounds, especially phenolic acids and their derivatives, and flavonoids in fruits and leaves of *L. intricatum* collected from Tunisia, such as chlorogenic, feruloylquinic, mono-caffeoylquinic, dicaffeoylquinic and *para*-coumaroylquinic acids, caffeoyl and di-caffeoyl putrescine, quercitrin, isoquercitrin, quercetin, rutin, rutinoside, di-rhamnoside, and kaempferol [16]. Similar results were obtained in leaf ethanol extracts of related species, namely *L. barbarum* and *L. chinensis* [42,43]. Overall, the phenolic compounds identified in *L. intricatum*, either in the present work or in previous reports, highlight the potential use of this species as a source of natural products with health improvement potential and different biotechnological applications, as, for example, in the food and cosmetic industries.

### 3.2. Antioxidant Activity

The highest RSA was obtained with the ethyl acetate and *n*-butanol fractions (Table 3). The crude ethanol extracts also showed a high RSA, which was significantly higher than that obtained with the used antioxidant standard (ascorbic acid), with EC_50_ values ranging from 13.59 to 77.16 µg/mL and the highest values being obtained with the ethanol extracts of roots. Conversely, the water fractions of leaves had the lowest capacity to scavenge the DPPH and ABTS^+^ radicals.

On the other hand, the ethyl acetate and *n*-butanol fractions of roots and leaves had a higher capacity to reduce iron (FRAP), but the ethyl acetate fraction of leaves was more efficient than other samples in terms of copper chelating potential (CCA). Samples were not active in the iron chelation assay (ICA) (Table 3). These results suggest that some extracts contain compounds with copper chelating activity, and that these compounds may have a phenolic nature. To the best of our knowledge, there were no previous reports regarding the copper chelating potential of *L. intricatum*.

Samples had a high RSA, which was higher in the crude ethanol extract from roots, when compared to its leaf’s counterpart, and had a significant capacity to reduce iron, like previous findings in a related species, *L. europaeum* [11]. The RSA and iron reducing capacity were higher than those reported for a methanol extract from leaves and fruits of the same species collected in Tunisia [16], which may be related with different factors known to affect the synthesis of secondary metabolites and, consequently, the biological properties of obtained extracts, including different sites of collection and methods of extraction. The values of RSA obtained in the present study were like those obtained with ethanol extracts from the leaves of *L. barbarum* and *L. chinense* [43], while the capacity to reduce iron of the ethyl acetate extract was similar to that reported by Yan et al. [44] for leaves of *L. barbarum*. In leaves, the RSA, iron reducing, and copper chelating properties were higher in the ethyl acetate and *n*-butanol fractions, which could be linked to the enrichment in phenolic content of those samples, since it is known that phenolics are able to quench free radicals by forming resonance-stabilized phenoxyl radicals [45]. The ethyl acetate fractions generally showed higher RSA, which might be due to the presence of semi-polar molecules, including flavonoids (Table 1). These results agree with others reporting that ethyl acetate was more effective for extracting antioxidants from other plant species, including *Sasa quelpaertensis* and *Pistacia atlantica* subsp. *atlantica* [46,47]. The root and leaf extracts also had a considerable iron reducing capacity, indicating that they have effective electron donors capable of reducing oxidized intermediates of lipid peroxidation [48]. Interestingly, in the present study, no capability to chelate iron was detected. It has been suggested that the iron chelating activity depends on the presence of catechol groups, which seem to be mostly responsible for metal chelating [45]. Therefore, our results might indicate that the phenolics present in the extracts have few catechol groups in their structures.

Phenolic compounds have a recognized strong antioxidant capacity [49]. In this sense, we can suggest that the antioxidant activity of *L. intricatum* most likely reflects its high phenolic content. Nonetheless, the detected phenolic compounds may contribute to the *L. intricatum* antioxidant capacity through addictive and/or synergistic effects [50]. Furthermore, differences between the phenolic composition and content of root and leaf extracts can be responsible for their different behaviours against the various oxidative agents, since detected compounds can have distinct activities towards the same oxidant. For instance, phenolic acids present in the roots and leaves of *L. intricatum* extracts, namely gallic, gentisic, ferulic, and *trans*-cinnamic acids, are excellent RSA, and they may be associated with the increased activity of these extracts. Gallate and dihydroxy groups can prevent metal-induced free radicals’ formation through copper chelation, which leads to inactive complexes formation [50]. In the same way, samples were not able to chelate iron, possibly due to a differential selectivity of the antioxidants towards the several oxidising agents [50,51]. From the present results, it is clear that extracts of *L. intricatum*, especially those from roots, contain molecules not only able to scavenge free radicals, namely DPPH and ABTS^+^, but also to reduce Fe^3+^ and to chelate copper; thus, they may be useful in the prevention of oxidative-stress diseases, including, for example, neurodegeneration, diabetes, and skin disorders [52].

### 3.3. Enzymatic Inhibitory Properties

The extracts were further evaluated for their capacity to inhibit enzymes implicated in the onset of human diseases, including neurodegeneration, T2DM, obesity/acne, and hyperpigmentation/food oxidation, and results are summarized in Table 4. Only the chloroform and the ethyl acetate root fractions significantly inhibited AChE, while none of the extracts were able to considerably inhibit BuChE (Table 4). To the best of our knowledge, there is no published data regarding the cholinesterase inhibitory activity of *L. intricatum* or other neuroprotective properties. A higher inhibitory capacity towards AChE (IC_50_ = 92.63 µg/mL) was previously reported for the *n*-butanol fraction obtained from an ethanol root extract of *L. europaeum* [11]. Such results were in accordance with previous studies of Mocan et al. [53], who observed lower values in terms of cholinesterase inhibition for methanol/water (70:30, *v/v*) leaf extracts of *L. barbarum*. Interestingly, the *n*-butanol fraction and crude ethanol extract from roots, and the ethyl acetate fraction from leaves, were able to inhibit α-glucosidase, which were significantly higher than that obtained with the positive control, acarbose. No information was found in the literature regarding the α-glucosidase inhibitory activity of *L. intricatum*. The results obtained in this work are in accordance with those reported in a previous one targeting *L. europaeum,* where the root extracts displayed a high inhibitory capacity towards that enzyme [11]. In another study, methanol leaf extracts of *L. chinense* were also found to be effective against α-glucosidase activity [54]. The higher activity observed in the polar extracts, i.e., *n*-butanol and ethanol, could be due to their higher phenolic content. Similar results were obtained by Custódio et al. [55], who reported that extracts made from *Quercus suber* L., with the highest phenolic content, also displayed the maximum α-glucosidase inhibition. It is well established that phenolic compounds play an important role in modulating glucosidase activities and, therefore, can contribute to the management of T2DM [55,56]. The present results suggest that roots of *L. intricatum* contain molecules capable of inhibiting the dietary carbohydrate digestive enzyme and AChE, which may be useful for the control of glucose levels in T2DM patients and for the treatment of AD through modulation of the neurotransmitter acetylcholine in the brain. In addition, the results also suggest that the highest AChE and α-glucosidase inhibitory activities displayed by some extracts may be related with the identified compounds. In fact, previous studies have demonstrated or reviewed these inhibitory activities for gallic acid (**1**), catechin (**3**), rutin (**5**), and quercetin (**7**) [57,58,59]. However, we cannot discard both a synergistic effect and the activity of other compounds not identified in the samples. None of the extracts were active against lipase. However, they were able to inhibit tyrosinase and the inhibitory activity of *n*-butanol, and water fractions from roots were higher than that of the positive control, arbutin (Table 4). Although no reports were found regarding the tyrosinase inhibition of *L. intricatum* extracts, this capacity was already reported for root extracts of a related species, *L. chinense* [60]. The stronger tyrosinase inhibition capacity exhibited by the root extracts may be related to some identified compounds, namely gallic **(1)** and gentisic **(2)** acids (Figure 1), which are tyrosinase inhibitors [61,62]. The present results encourage further work aiming to deepen knowledge on the potential use of *L. intricatum* as a source of skin whitening products and food additives, which could be of interest for the food, cosmetic, and pharmaceutical industries. In fact, besides its involvement in melanin production, tyrosinase is also related with enzymatic browning, which is a major problem of fresh-cut fruits, and results from oxidation reactions with several enzymes and leads to modifications in the appearance of the nutritional value of food stuffs. Sulfiting agents are the most frequently used anti-browning products but have adverse health effects. Thus, safer anti-browning additives are much needed, and several natural products were already identified, including polyphenol-rich extracts [63]. Of note is the fact that, although the ethanol extract was not active in some assays, namely AChE, BuChE, lipase, and tyrosinase, the obtained fractions displayed some inhibition, allowing for the calculation of IC_50_ values (Table 4). This can be explained by an accumulation of molecules with enzymatic inhibition properties because of the fractionating process. In the same way, Bendjedou et al. [11] investigated the root and leaf extracts of *L. europaeum* for in vitro enzyme inhibitory activities. Obtained fractions displayed relevant inhibitory activity towards AChE, BuChE, and urease, while the crude ethanol extract was not active. These findings correlate with the results of the present study. A more detailed analysis of the phytochemical profile of the active fractions is needed to identify molecules with the antienzyme actions observed in this study.

## 4. Conclusions

This study reports, for the first time, that extracts from *L. intricatum* roots have radical scavenging, ferric reducing, and metal chelating activities, coupled with enzyme inhibitory activity towards AChE, α-glucosidase, and tyrosinase. These bioactivities may be related to the high abundance of total phenolics in the extracts and to some identified molecules, such as gallic acid (**1**), catechin (**3**), rutin (**5**), and quercetin (**7**). Our results are generally similar to those obtained with well-studied *Lycium* species, such as *L. barbarum* and *L. chinense*, and suggest that roots and leaves of *L. intricatum* could be considered a source of innovative herbal products, with applications in the food and pharmaceutical industries, with particular interest in the prevention of oxidative stress, neurological diseases, diabetes, and skin disorders. Additional experiments are needed to identify and characterize the bioactive compounds present in the extracts, namely through a bioguided fractionation and isolation of pure compounds. Our results could be used to the valorisation of this promising species.

## Figures and Tables

**Table 1:** Extraction yields and total phenolics, flavonoids, and hydrolysable tannins content of ethanol extracts from *L. intricatum* and obtained fractions.

	Yield (%)		TPC (mg GAE/g DE)		TFC (mg CE/g DE)		THTC (mg TAE/g DE)	
Extract/Fraction	Roots	Leaves	Roots	Leaves	Roots	Leaves	Roots	Leaves
Ethanol	1.805	11.070	119.19 ± 4.23 ^a^	53.39 ± 3.94 ^a^	63.13 ± 0.47 ^a^	21.86 ± 0.25 ^a^	53.98 ± 4.39^a^	n.d.
Chloroform	0.024	0.118	118.02 ± 2.06 ^a^	124.18 ± 4.54 ^a,c^	13.01 ± 0.16 ^b^	22.38 ± 0.51 ^a^	n.d.	n.d.
Ethyl acetate	0.021	0.235	281.62 ± 26.64 ^b^	268.57 ± 40.96 ^b,d^	141.31 ± 21.36 ^c^	185.51 ± 14.08 ^b^	472.01 ± 39.95 ^b^	282.01 ± 30.62
*n*-butanol	0.122	1.520	266.56 ± 3.47 ^b^	215.69 ± 19.48 ^c,d^	70.81 ± 4.85 ^a^	75.32 ± 0.61 ^c^	373.34 ± 27.48 ^b^	n.d.
Water	0.463	3.873	46.73 ± 4.66 ^c^	35.09 ± 2.08 ^a^	47.86 ± 0.26 ^a,b^	20.55 ± 0.62 ^a^	71.40 ± 2.00 ^a^	n.d.

n.d.: not determined. Values represent the mean ± standard error of the mean (SEM) of triplicate samples. In the same column, values followed by different letters are significantly different according to the Tukey and Student–Newman–Keuls multiple range tests (*p* < 0.05). TPC: total phenolics content; TFC: total flavonoids content; THTC: total hydrolysable tannins content.

**Table 2:** Molecules identified in *L. intricatum* extracts.

Chemical Compound	Chemical Structure	Formula	Classification	Organ	Biological Properties	Reference
Gallic acid (1)	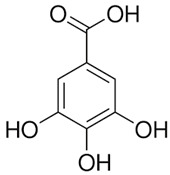	C_6_H_2_(OH)_3_CO_2_H	Phenolic acid	Leaves and roots	Antioxidant, antimicrobial, anti-tumor, anti-inflammatory, anti-melanogenic, anti-viral, anti-allergic, neuroprotective, nephroprotective, hepatoprotective.	[34]
Gentisic acid (2)	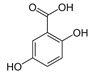	C_6_H_3_(CO_2_H)(OH)_2_	Phenolic acid	Leaves and roots	Anti-inflammatory, anti-genotoxic, hepatoprotective, neuroprotective, antimicrobial, antioxidant.	[35]
Catechin (3)	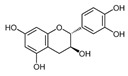	C_15_H_14_O_6_	Polyphenol (flavonoid)	Leaves	Antioxidant, UV protection, antimicrobial, anti-allergenic, anti-inflammatory, anti-viral, anti-cancer, activation of skin barrier passage, promotion of cell activity.	[36]
*p*-Coumaric acid (4)	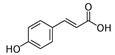	HOC_6_H_4_CH=CHCO_2_H	Phenolic acid	Roots	Antioxidant, anti-inflammatory, analgesic, antimicrobial.	[37]
Rutin (5)	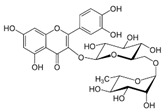	C_27_H_30_O_16_	Polyphenol (flavonoid)	Leaves	Antioxidant, cytoprotective, vasoprotective, anti-carcinogenic, neuroprotective, cardioprotective.	[38]
Gallocatechin gallate (6)	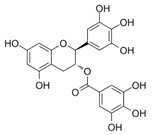	C_22_H_18_O_11_	Polyphenol (flavonoid)	Leaves	Antioxidant, UV protection, antimicrobial, anti-allergenic, anti-inflammatory, anti-viral, anti-cancer, activation of skin barrier passage, promotion of cell activity.	[36]
Quercetin (7)	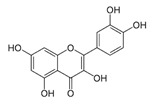	C_15_H_10_O_7_	Polyphenol (flavonoid)	Leaves	Antioxidant, radical-scavenging, anti-inflammatory, anti-bacterial, anti-viral, gastroprotective, immune-modulatory.	[39]
Ferulic acid (8)	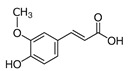	(CH_3_O)HOC_6_H_3_CH=CHCO_2_H	Phenolic acid	Leaves and roots	Anti-inflammatory, antioxidant, antimicrobial, anti-cancer, anti-diabetic.	[40]
*trans*-cinnamic acid (9)	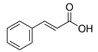	C_6_H_5_CH=CHCO_2_H	Phenolic acid	Leaves and roots	Anti-tumoral, anti-bacterial, anti-diabetic, neuroprotective.	[41]

The number in brackets refers to the peaks in the chromatograms of Figure 1 and Figure 2.

**Table 3:** Radical-scavenging activity on DPPH and ABTS^+^ radicals, ferric reducing antioxidant power (FRAP), and metal-chelating activities on iron (ICA) and copper (CCA) of ethanol extracts from *L. intricatum* and obtained fractions. Results are expressed as EC_50_ values (µg/mL).

	DPPH		ABTS		FRAP		ICA		CCA	
Extract/Fraction/ Standard	Roots	Leaves	Roots	Leaves	Roots	Leaves	Roots	Leaves	Roots	Leaves
Ethanol	43.92 ± 0.21 ^a^	77.16 ± 0.94 ^a^	13.59 ± 0.24 ^a^	45.30 ± 0.47 ^a^	175.06 ± 4.21 ^a^	460.86 ± 15.91 ^a^	n.a.	n.a.	2657.33 ± 45.36 ^a^	n.a.
Chloroform	95.74 ± 0.91 ^b^	71.84 ± 2.24 ^a^	14.69 ± 0.24 ^a^	13.59 ± 0.42 ^b^	599.55 ± 39.96 ^b^	187.30 ± 15.91 ^b^	n.a.	n.a.	n.a.	n.a.
Ethyl acetate	61.11 ± 2.09 ^c^	20.42 ± 0.36 ^b^	12.32 ± 0.17 ^a^	10.32 ± 0.27 ^b^	594.12 ± 20.02 ^b^	181.00 ± 11.04 ^b^	n.a.	n.a.	n.a.	1555.66 ± 13.83 ^a^
*n*-butanol	42.25 ± 0.54 ^a^	46.48 ± 0.43 ^c^	12.57 ± 0.26 ^a^	21.24 ± 0.63 ^b^	240.47 ± 17.66 ^a^	359.36 ± 11.06 ^c^	n.a.	n.a.	n.a.	3323.66 ± 28.38 ^b^
Water	69.89 ± 1.97 ^c^	173.76 ± 1.50 ^d^	39.84 ± 0.59 ^b^	129.71 ± 1.88 ^c^	859.66 ± 28.71 ^c^	1519.33 ± 34.16 ^d^	n.a.	n.a.	n.a.	n.a.
BHT *	n.t.	n.t.	n.t.	n.t.	-	-	n.t.	n.t.	n.t.	n.t.
Ascorbic acid *	125.95 ± 4.65 ^d^	125.95 ± 4.65 ^e^	218.31 ± 7.39 ^c^	218.31 ± 7.39 ^d^	n.t.	n.t.	n.t.	n.t.	n.t.	n.t.
EDTA *	n.t.	n.t.	n.t.	n.t.	n.t.	n.t.	33.04 ± 1.60	33.04 ± 1.60	120.60 ± 2.11 ^b^	120.60 ± 2.11 ^c^

* Positive controls; n.t.: not tested; n.a.: not active (EC_50_ value not reached). Values represent the mean ± standard error of the mean (SEM) of triplicate samples. In the same column, values followed by different letters are significantly different according to the Tukey and Student–Newman–Keuls multiple range tests (*p* < 0.05).

**Table 4:** Enzymatic inhibitory properties of ethanol extracts from *L. intricatum* and obtained fractions. Results are expressed as IC_50_ values (µg/mL).

	Acetylcholinesterase		Butyrylcholinesterase		Glucosidase		Lipase		Tyrosinase	
Extract/Fraction/Standard	Roots	Leaves	Roots	Leaves	Roots	Leaves	Roots	Leaves	Roots	Leaves
Ethanol	n.a.	n.a.	n.a.	n.a.	944.85 ± 14.17 ^a^	n.a.	n.a.	n.a.	n.a.	n.a.
Chloroform	790.93 ± 43.97 ^a^	n.a.	n.a.	n.a.	n.a.	n.a.	n.a.	n.a.	n.a.	n.a.
Ethyl acetate	998.83 ± 33.87 ^b^	n.a.	n.a.	n.a.	n.a.	1890.66 ± 56.62 ^a^	n.a.	n.a.	3549.75 ± 199.06 ^a^	n.a.
*n*-butanol	n.a.	n.a.	n.a.	n.a.	733.20 ± 25.80 ^a^	n.a.	n.a.	n.a.	162.90 ± 20.05 ^b^	3808.00 ± 413.33 ^a^
Water	n.a.	n.a.	n.a.	n.a.	n.a.	n.a.	n.a.	n.a.	274.07 ± 8.09 ^b^	n.a.
Galantamine *	7.8 ± 0.44 ^c^	7.8 ± 0.44	320 ± 30	320 ± 30	n.t.	n.t.	n.t.	n.t.	n.t.	n.t.
Acarbose *	n.t.	n.t.	n.t.	n.t.	2955.00 ± 158.25 ^b^	2955.00 ± 158.25 ^b^	n.t.	n.t.	n.t.	n.t.
Orlistat *	n.t.	n.t.	n.t.	n.t.	n.t.	n.t.	120 ± 10	120 ± 10	n.t.	n.t.
Arbutin *	n.t.	n.t.	n.t.	n.t.	n.t.	n.t.	n.t.	n.t.	409.08 ± 1.97 ^b^	409.08 ± 1.97 ^b^

* Positive controls; n.t.: not tested; n.a.: not active (IC_50_ value not reached). Values represent the mean ± standard error of the mean (SEM) of triplicate samples. In the same column, values followed by different letters are significantly different according to the Tukey and Student–Newman–Keuls multiple range tests (*p <* 0.05).

## Data Availability

Not applicable.
